# NRG Oncology/NSABP B-47 menstrual history study: impact of adjuvant chemotherapy with and without trastuzumab

**DOI:** 10.1038/s41523-021-00264-2

**Published:** 2021-05-20

**Authors:** Patricia A. Ganz, Reena S. Cecchini, Louis Fehrenbacher, Charles E. Geyer, Priya Rastogi, John P. Crown, Michael P. Thirlwell, David M. Ellison, Jean-Francois Boileau, Patrick J. Flynn, Jong-Hyeon Jeong, Eleftherios P. Mamounas, Norman Wolmark

**Affiliations:** 1 NSABP/NRG Oncology, Pittsburgh, PA USA; 2 University of California at Los Angeles, Los Angeles, CA USA; 3 University of Pittsburgh, Pittsburgh, PA USA; 4 Kaiser Permanente Oncology Clinical Trials Northern CA, Novato, CA USA; 5 Houston Methodist Cancer Center, Houston, TX USA; 6 The University of Pittsburgh Cancer Institute, Pittsburgh, PA USA; 7 Irish Cooperative Oncology Research Group/Cancer Trials Ireland, Dublin, Ireland; 8 Montréal General Hospital, McGill University Health Centre, Montréal, Canada; 9 Roper St Francis Healthcare, Charleston, SC USA; 10 Jewish General Hospital Segal Cancer Centre McGill University, Montréal, Québec, Canada; 11 Minnesota Community Oncology Research Consortium (MSORC), Stone Lake, WI USA; 12 Orlando Health UF Health Cancer Center, Orlando, FL USA

**Keywords:** Breast cancer, Cancer screening

## Abstract

The NRG Oncology/NSABP B-47 menstrual history (MH) study examined trastuzumab effects on menstrual status and associated circulating reproductive hormones. MH was evaluated by questions related to hysterectomy, oophorectomy, and reported menstrual changes. Pre/perimenopausal women were assessed at entry, 3, 6, 12, 18, 24, 30, and 36 months. Consenting women had estradiol and FSH measurement at entry, 3, 6, 12, 18, and 24 months. Logistic regression determined predictors of amenorrhea and hormone levels at 12, 24, and 36 months. Between 2/8/2011 and 2/10/2015, 3270 women with node-positive/high-risk node-negative HER2-low breast cancer were enrolled. There were 1,458 women enrolled in the MH study; 1231 consented to baseline blood samples. Trastuzumab did not contribute to a higher amenorrhea rate. Amenorrhea predictors were consistent with earlier studies; however, to our knowledge, this is the largest prospective study to include serial reproductive hormone measurements to 24 months and clinical amenorrhea reports to 36 months. These data can help to counsel patients regarding premature menopause risk.

## Introduction

NRG Oncology/NSABP B‐47 is a phase III, multicenter, randomized adjuvant therapy trial designed to evaluate the addition of trastuzumab to adjuvant chemotherapy in patients with HER2-low breast cancer. The primary endpoint was invasive disease‐free survival (IDFS) and clinical results showed no benefit from the addition of trastuzumab to chemotherapy in this population^1^. Because the impact of trastuzumab on menstrual function was unknown at the time of protocol design, a secondary endpoint focused on menstrual health. The primary aims of the menstrual history (MH) sub-study were to assemble an observational cohort of pre- and perimenopausal women to evaluate the effect of chemotherapy with or without trastuzumab on treatment-related amenorrhea (TRA) and the associations between TRA and circulating reproductive hormone levels. Secondary exploratory aims examined associations between chemotherapy regimen, TRA, and IDFS benefit in premenopausal women. Here we report on these outcomes, as well as medical and demographic predictors of TRA.

## Results

### Patients

**Fig. 1:**
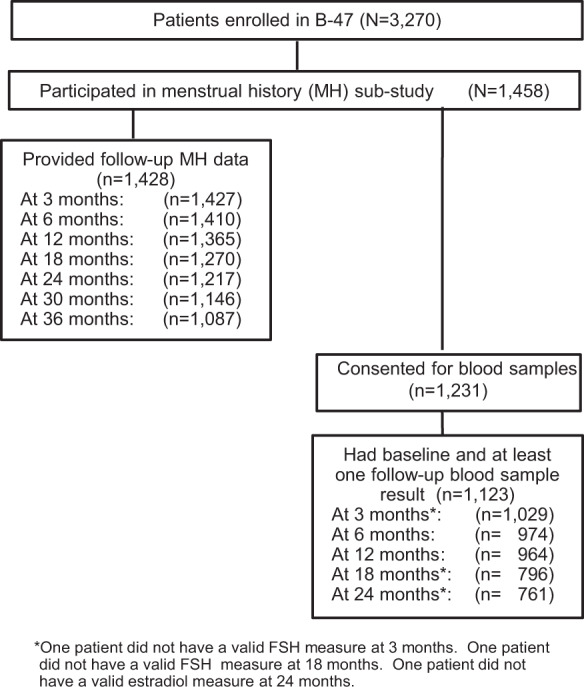
CONSORT Diagram. NSABP B-47 Menstrual History Sub-study.

The B-47 protocol entered 3270 patients between February 8, 2011 and February 10, 2015. The MH sub-study enrolled 1458 eligible women, and 1231 consented to blood sample collection. This report uses data collected through June 30, 2019. Figure 1 shows the study CONSORT diagram. Both baseline and at least one follow-up MH form were available for 1428 patients. There was high compliance with form submission across the 36 months. Both baseline and at least one follow‐up blood hormone measure were available for 1123 patients.

The mean age of participants was 44.1 years. Table 1 shows the distribution and tumor characteristics by randomized treatment groups and by chemotherapy regimens for all analyzed MH patients. There were no differences in characteristics between those assigned to trastuzumab vs no trastuzumab. Anthracycline regimen recipients were statistically significantly younger, had more positive nodes, were more likely to have hormone receptor (HR)-negative cancer, a higher histologic grade, and to have had a mastectomy, compared to those treated with the non-anthracycline regimen. Participants in the MH sub-study were significantly younger (*P* < 0.0001); more likely to be non-White (*P* = 0.003); Hispanic (*P* = 0.01); hormone-receptor positive (<0.0001); and to have received doxorubicin and cyclophosphamide (AC) followed by weekly paclitaxel (WP) for 12 weeks (*P* < 0.0001) as compared to the B-47 participants not in the MH sub-study (data not shown).

**Table 1:** Patient and tumor characteristics by treatment group and intended chemotherapy regimen for analyzed patients: NSABP B-47.

Characteristic	All		By randomized treatment group					By intended chemotherapy regimen				
			Chemo alone		Chemo + Trast		*P*^1^	AC → WP		TC		*P*^1^
	No.	(%)	No.	(%)	No.	(%)		No.	(%)	No.	(%)	
Age (yrs)												
≤40	380	(26.6)	191	(26.9)	189	(26.3)	0.76	302	(30.5)	78	(17.8)	<0.0001
41–45	397	(27.8)	191	(26.9)	206	(28.7)		276	(27.9)	121	(27.7)	
46–50	436	(30.5)	215	(30.3)	221	(30.8)		290	(29.3)	146	(33.4)	
>50	215	(15.1)	113	(15.9)	102	(14.2)		123	(12.4)	92	(21.1)	
Race												
White	1164	(81.5)	580	(81.7)	584	(81.3)	0.75^2^	807	(81.4)	357	(81.7)	0.27^2^
Black	136	(9.5)	66	(9.3)	70	(9.7)		91	(9.2)	45	(10.3)	
Native Hawaiian/Other Pacific Islander	6	(0.4)	3	(0.4)	3	(0.4)		5	(0.5)	1	(0.2)	
Asian	72	(5.0)	38	(5.4)	34	(4.7)		55	(5.5)	17	(3.9)	
American Indian or Alaska Native	8	(0.6)	5	(0.7)	3	(0.4)		5	(0.5)	3	(0.7)	
Multi-racial	8	(0.6)	2	(0.3)	6	(0.8)		8	(0.8)	0	(0.0)	
Unknown	34	(2.4)	16	(2.3)	18	(2.5)		20	(2.0)	14	(3.2)	
Ethnicity												
Not Hispanic or Latino	1292	(90.5)	632	(89.0)	660	(91.9)	0.34	901	(90.9)	391	(89.5)	0.43
Hispanic or Latino	106	(7.4)	57	(8.0)	49	(6.8)		70	(7.1)	36	(8.2)	
Unknown	30	(2.1)	21	(3.0)	9	(1.3)		20	(2.0)	10	(2.3)	
IHC Score												
1+	836	(58.5)	415	(58.5)	421	(58.6)	0.94	590	(59.5)	246	(56.3)	0.25
2+	592	(41.5)	295	(41.5)	297	(41.4)		401	(40.5)	191	(43.7)	
Number of positive nodes												
0	281	(19.7)	153	(21.5)	128	(17.8)	0.34	167	(16.9)	114	(26.1)	<0.0001
1–3	776	(54.3)	377	(53.1)	399	(55.6)		519	(52.4)	257	(58.8)	
4–9	270	(18.9)	129	(18.2)	141	(19.6)		220	(22.2)	50	(11.4)	
10+	101	(7.1)	51	(7.2)	50	(7.0)		85	(8.6)	16	(3.7)	
Hormone-receptor status												
ER and PgR negative	199	(13.9)	95	(13.4)	104	(14.5)	0.55	157	(15.8)	42	(9.6)	0.002
ER and/or PgR positive	1229	(86.1)	615	(86.6)	614	(85.5)		834	(84.2)	395	(90.4)	
Intended chemotherapy regimen												
AC → WP	991	(69.4)	502	(70.7)	489	(68.1)	0.29					
TC	437	(30.6)	208	(29.3)	229	(31.9)						
Histologic grade												
Low	120	(8.4)	58	(8.2)	62	(8.6)	0.22	60	(6.1)	60	(13.7)	<0.00001
Intermediate	603	(42.2)	285	(40.1)	318	(44.3)		416	(42.0)	187	(42.8)	
High	704	(49.3)	366	(51.5)	338	(47.1)		514	(51.9)	190	(43.5)	
Unknown	1	(0.1)	1	(0.1)	0	(0.0)		1	(0.1)	0	(0.0)	
Type of surgery												
Lumpectomy	564	(39.5)	289	(40.7)	275	(38.3)	0.64	360	(36.3)	204	(46.7)	0.0003
Mastectomy	810	(56.7)	394	(55.5)	416	(57.9)		597	(60.2)	213	(48.7)	
Both	54	(3.8)	27	(3.8)	27	(3.8)		34	(3.4)	20	(4.6)	
Body mass index												
<18.5	18	(1.3)	8	(1.1)	10	(1.4)	0.77	12	(1.2)	6	(1.4)	0.18
18.5–24.9	487	(34.1)	234	(33.0)	253	(35.2)		348	(35.1)	139	(31.8)	
25.0–29.9	423	(29.6)	216	(30.4)	207	(28.8)		302	(30.5)	121	(27.7)	
≥30.0	500	(35.0)	252	(35.5)	248	(34.5)		329	(33.2)	171	(39.1)	
All	1428	(100.0)	710	(100.0)	718	(100.0)		991	(100.0)	437	(100.0)	

*AC→WP* doxorubicin and cyclophosphamide followed by weekly paclitaxel for 12 weeks, *ER* estrogen receptor, *IHC* immunohistochemistry, *PgR* progesterone receptor, *TC* docetaxel plus cyclophosphamide, *Trast* trastuzumab.

^1^*P* values are based on the Chi-square test unless otherwise indicated. All unknown values are excluded.

^2^Fisher’s exact test.

### Amenorrhea

We hypothesized that the cessation of menses for at least 6 months’ duration would be associated with postmenopausal estradiol levels and elevated follicle-stimulating hormone (FSH) levels. However, the rate of agreement between amenorrhea status and estradiol and FSH levels at both 12 and 24 months was poor (Supplementary Table 1). At 12 months, the kappa statistics were 0.36, 0.11, and 0.09, for estradiol, FSH, and the combined indicator for estradiol and FSH, respectively. At 24 months, the kappa statistics were 0.44, 0.08, and 0.08, respectively. Of the 648 women who had data available for MH, estradiol, and FSH at 24 months, there were 410 with TRA, however, hormone levels were in the premenopausal range (estradiol ≥ 20 pg/mL and FSH < 50 mIU/mL). Of those, 360 (88%) reported the use of tamoxifen.

We also hypothesized that trastuzumab would have no effect on the rate of cessation of menses for ≥6 months. As shown in Supplementary Fig. 1, there were no differences in TRA rates between the trastuzumab and non-trastuzumab intention‐to‐treat groups. Furthermore, there were no statistically significant differences between treatment groups after adjusting for age at any of the three time points. At 12 months, 84.0% of patients in the trastuzumab group were amenorrheic vs 86.3% in the non-trastuzumab group (*P* = 0.20). Age-adjusted *P* values at 24 and 36 months were 0.81 and 0.83, respectively (Table 2).

**Table 2:** Results of logistic regression^a^ predicting amenorrhea status at 12, 24, and 36 months: NSABP B-47.

Characteristic	12 Months					24 Months					36 Months				
	No. of pts	% Amen	OR	(95% CI)	*P*	No. of pts	% Amen	OR	(95% CI)	*P*	No. of pts	% Amen	OR	(95% CI)	*P*
Age group (yrs)															
≤40	332	59.3	0.03	(0.01– 0.09)	<0.0001	128	46.0	0.03	(0.01– 0.06)	<0.0001	105	44.9	0.03	(0.01– 0.09)	<0.0001
41–45	347	89.6	0.19	(0.07– 0.53)		253	83.2	0.15	(0.06– 0.37)		224	84.2	0.19	(0.07– 0.53)	
46–50	390	96.9	0.68	(0.22– 2.13)		338	97.7	1.24	(0.40– 3.83)		309	96.3	0.68	(0.22– 2.13)	
>50	190	97.9	Ref			171	97.2	Ref			155	95.7	Ref		
Race															
White	1023	85.1	Ref		0.52	903	82.5	Ref		0.03	813	81.7	Ref		0.93
Black	120	85.8	1.35	(0.72– 2.52)		102	69.6	0.52	(0.30–0.92)		90	75.6	0.98	(0.52–1.84)	
Other	85	87.1	1.30	(0.63– 2.69)		77	74.0	0.59	(0.31–1.11)		64	78.1	0.87	(0.43–1.78)	
Ethnicity															
Not Hispanic or Latino	1137	85.8	Ref		0.21	998	81.1	Ref		0.83	894	81.4	Ref		0.59
Hispanic or Latino	96	76.0	0.68	(0.38– 1.23)		85	74.1	1.07	(0.56–2.04)		69	73.9	1.22	(0.60–2.48)	
Body mass index															
<18.5	16	93.8	3.48	(0.42– 28.95)	0.02	11	90.9	1.22	(0.13–11.75)	<0.0001	11	90.9	1.59	(0.17–14.88)	0.29
18.5–24.9	428	85.5	Ref			380	83.2	Ref			342	81.3	Ref		
25.0–29.9	375	86.7	1.19	(0.75– 1.90)		335	83.0	1.19	(0.73–1.94)		293	80.9	1.18	(0.72–1.93)	
≥30.0	440	83.2	0.63	(0.41– 0.97)		378	75.7	0.43	(0.28 –0.68)		337	79.5	0.75	(0.47–1.18)	
Treatment															
Chemo	640	86.3	Ref		0.20	566	80.2	Ref		0.81	504	80.6	Ref		0.83
Chemo + Trast	619	84.0	0.79	(0.55–1.13)		538	81.0	1.05	(0.73–1.51)		479	80.8	0.96	(0.65–1.41)	
Chemotherapy regimen															
AC → WP	874	84.0	Ref		0.50	768	79.0	Ref		0.60	683	80.1	Ref		0.06
TC	385	87.8	0.87	(0.58–1.31)		336	84.2	0.89	(0.59–1.36)		300	82.0	0.66	(0.43–1.01)	
IHC score															
1+	730	86.0	Ref		0.16	637	80.7	Ref		0.93	567	81.7	Ref		0.32
2+	529	83.9	0.77	(0.54–1.11)		467	80.5	1.02	(0.70–1.47)		416	79.3	0.82	(0.56–1.21)	
Number of positive nodes															
0	243	80.7	Ref		0.39	209	75.6	Ref		0.70	186	72.6	Ref		0.42
1–3	694	86.2	1.20	(0.75–1.89)		616	80.8	1.12	(0.70–1.82)		548	81.8	1.43	(0.88–2.34)	
4–9	244	88.5	1.30	(0.72–2.33)		212	84.0	1.07	(0.59–1.96)		188	84.6	1.21	(0.65–2.25)	
10+	78	79.5	0.70	(0.33–1.50)		67	83.6	1.73	(0.70–4.28)		61	83.6	1.86	(0.73–4.75)	
Hormone-receptor status															
ER and PgR negative	169	75.1	Ref		0.01	144	64.6	Ref		<0.0001	127	64.6	Ref		0.002
ER and/or PgR positive	1090	86.7	1.85	(1.16–2.96)		960	83.0	2.68	(1.65–4.36)		856	83.1	2.27	(1.37–3.77)	
Histologic grade															
Low	112	90.2	Ref		0.50	105	86.7	Ref		0.10	90	86.7	Ref		0.91
Intermediate	534	88.2	1.35	(0.64–2.82)		471	85.1	1.54	(0.75–3.18)		423	83.7	1.18	(0.55–2.53)	
High	612	81.7	1.09	(0.53–2.25)		528	75.4	1.02	(0.50–2.06)		470	76.8	1.12	(0.53–2.37)	
HR status/ET use															
HR−	172	76.2	Ref		0.02	146	65.1	Ref		<0.0001	129	65.1	Ref		0.003
HR+, none	43	69.8	0.86	(0.35–2.14)		18	55.6	1.2	(0.28–5.14)		15	66.7	2.29	(0.46–11.45)	
HR+, tamoxifen	931	86.5	1.75	(1.09–2.82)		843	82.1	2.54	(1.56–4.14)		763	82.0	2.14	(1.29–3.56)	
HR+, other	112	93.8	2.88	(1.10–7.50)		97	95.9	10.93	(3.11–38.37)		76	96.1	9.11	(2.18–38.13)	

*AC→WP* doxorubicin and cyclophosphamide followed by weekly paclitaxel for 12 weeks, *ER* estrogen receptor, *ET* endocrine therapy, *HR* hormone receptor, *IHC* immunohistochemistry, *PgR* progesterone receptor, *TC* docetaxel plus cyclophosphamide, *Trast* trastuzumab.

^a^Each characteristic was assessed univariably in separate logistic regression models, however, all except age group were adjusted for continuous age upon random assignment.

Table 2 also presents the results for other potential explanatory factors in predicting TRA at each time point. We first looked at age, confirming that it was a statistically significant predictor of TRA at 12, 24, and 36 months. After adjusting for age, there was no difference in amenorrhea status between groups defined by ethnicity, chemotherapy regimen, IHC score, number of positive nodes, or histologic grade. There was a statistically significant difference in race at 24 months with Black women having a decreased likelihood of TRA compared to White women. Body mass index (BMI) was a statistically significant predictor of TRA at 12 and 24 months, in that obese women were less likely to become amenorrheic. HR status/ET use was also a statistically significant predictor for TRA at each time point. Specifically, compared to HR‐negative patients, those who were HR‐positive and had reported use of tamoxifen or other endocrine therapy (ET) were more likely to be amenorrheic.

At each time point, we investigated a multivariable model with potential explanatory variables using backward elimination. For those models, the HR status/ET use was examined instead of HR status alone. At 12 and 24 months, all variables dropped out of the model except age, BMI, and HR status/ET use. However, at 36 months, chemotherapy regimen was a statistically significant predictor with age and HR status/ET. Supplementary Table 2 provides results of this multivariable model, showing that the non-anthracycline regimen had lower odds of TRA (OR = 0.60, 95% CI = 0.39–0.94; *P* = 0.02).

### Estradiol and FSH

**Fig. 2:**
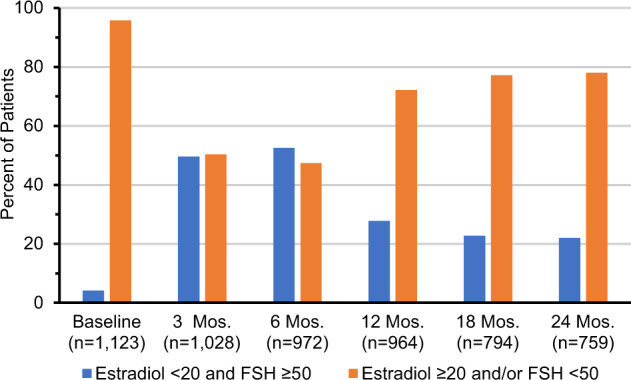
Distribution of Combined Estradiol and FSH levels over Time.

The distribution of estradiol and FSH levels categorized into estradiol <20 pg/mL and FSH ≥ 50 mIU/mL versus estradiol ≥20 pg/mL and/or FSH < 50 mIU/mL across all time points is presented in Fig. 2. The baseline percent of patients with estradiol and FSH levels in the postmenopausal range (estradiol < 20 pg/mL and FSH ≥ 50 mIU/mL) was 4.2%. This increased to 49.6% (3 months) and 52.6% (6 months), then dropped to 27.8% (12 months) and 22% (24 months). Supplementary Table 3 presents logistic regression results for predicting low estradiol and high FSH. At 6 months, there were statistically significant differences between chemotherapy regimens (*P* = 0.008), IHC scores (*P* = 0.004), and BMI groups (*P* < 0.0001). Obese women were less likely to have estradiol and FSH levels in the postmenopausal range as compared to women in the normal BMI range. BMI remained a statistically significant predictor at 12 months, but not at 24 months. Histologic grade was a statistically significant predictor at both 12 and 24 months (*P* = 0.03 at both time points), with increased odds of postmenopausal hormone levels in those with high vs low grade. ET use during the first 6 months after randomization was not available, but HR status/ET use was a statistically significant predictor at 12 and 24 months, with overall *P* values <0.0001 at both time points. When considering a multivariable model at 24 months, only age and HR status/ET use (controlling for baseline estradiol and FSH level), remained statistically significant. Patients with HR‐positive cancer with reported tamoxifen use were less likely to have estradiol and FSH levels in the postmenopausal range as compared to HR‐negative patients (OR = 0.24, 95% CI = 0.15–0.39).

**Fig. 3:**
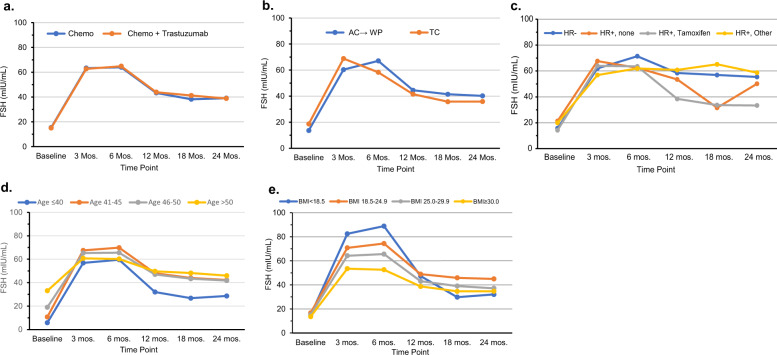
Mean Follicle-stimulating Hormone (FSH) Levels Respectively over Time in NSABP B-47. Levels are presented by: treatment group (**a**), chemotherapy regimen (**b**), HR status/endocrine therapy use (**c**), age group (**d**), and BMI (**e**). A higher FSH level indicates a late premenopausal/postmenopausal range. Adjusted least-square means for time points 3 months and beyond were obtained from a mixed model for repeated measures analysis of the FSH level. The model includes age, FSH baseline level, the variable of interest, time, and variable-by-time interaction.

Figure 3 shows the mean FSH levels respectively over time by treatment group (a), chemotherapy regimen (b), HR status/ET use (c), age group (d), and BMI (e). Treatment groups did not differ over time. Overall, chemotherapy regimen did not reach statistical significance (*P* = 0.08); however, the plot shows a qualitative interaction over time (*P* < 0.0001). HR status/ET use (defined as ever used in 24 months) was statistically significant and varied over time (*P* < 0.0001) with the tamoxifen group having the lowest FSH levels at 12 and 24 months. As expected, there was a difference in age with younger women having lower FSH levels. BMI was also statistically significant, and the magnitude of the difference varied over time (*P* < 0.0001). Obese women consistently had lower FSH levels than those with normal BMIs. Underweight women had higher FSH levels early on, but lower levels by 24 months. However, it should be noted that due to the low number of women in the underweight category, these estimates are inconclusive.

### Persistent amenorrhea

An estimate of persistent amenorrhea was also of interest. Supplementary Table 4 shows the distribution of amenorrhea status at 24 and 36 months among those amenorrheic at 12 months. Among those amenorrheic at 12 months, 79.0% remained amenorrheic at 24 months, and 70.0% remained amenorrheic at 36 months. Persistent amenorrhea in relation to postmenopausal levels of FSH and estradiol are also presented. Among those who were amenorrheic at 12 months with high FSH and low estradiol at 12 months, 84.1% and 74.8% remained amenorrheic at 24 and 36 months, respectively.

### Amenorrhea and IDFS

Although underpowered in this trial subgroup, we investigated the prognostic effect of TRA using each patient’s amenorrhea status at 12 months. There were 1249 patients in the landmark population, and median follow-up time from 12 months post-randomization was 4.2 years, with 76 IDFS events. Five-year IDFS was 93.6% and 92.5% in the amenorrhea and no amenorrhea groups, respectively, HR 0.67 (95% CI = 0.36–1.28), *P* = 0.23 (Supplementary Fig. 2).

## Discussion

In this large, prospective, observational study of pre- and perimenopausal women receiving contemporary chemotherapy regimens, we found no difference in TRA between the trastuzumab and non-trastuzumab groups. Blood specimens were also collected to measure menopausal reproductive hormones to evaluate their usefulness in tracking the onset of menopause, along with clinical documentation of menstrual status. Using a combination of estradiol levels in the postmenopausal range and elevated levels of FSH, at 6 months after random assignment, more than half of the women had postmenopausal hormone profiles; however, by 24 months reproductive hormone levels had improved such that 22% had postmenopausal levels. Among those with premenopausal hormone levels and amenorrhea, ET with tamoxifen was likely a contributing factor to persistent amenorrhea.

Adjuvant chemotherapy has known risks for the development of TRA in premenopausal women^2,3^. Although chemotherapy treatment regimen was selected by investigators from two established regimens, and was equally distributed across the two treatment arms in the entire B-47 trial^1^, among the younger women in the MH sub-study, the anthracycline-containing regimen was more often selected (69%). In addition, the anthracycline-containing regimen was more likely to be chosen for women with HR-negative tumors, positive nodes, and high-grade tumors (Table 1). Thus, in addition to randomized treatment group, intended chemotherapy regimen was also an important exploratory variable.

Only a few studies have examined the impact of adjuvant HER2-directed therapy on menstrual status^4–6^. Lambertini et al.^4^ recently reported on the impact of adjuvant lapatinib and/or trastuzumab (four treatment arms) in the BIG 2-06 trial, in which menopausal status data were collected at randomization and at the 37-week visit. TRA was described in 2682 premenopausal women, and the impact of amenorrhea on disease-free and overall survival were examined using landmark and time-dependent modeling. Prior to randomization, patients had received heterogeneous adjuvant chemotherapy regimens that were included in the stratification. In addition, diverse ET treatments were received in women with HR-positive tumors during the course of the HER2-directed therapy. To be included in the premenopausal sample, patients had to have had a menstrual period within 6 months of randomization or be <50 years. In the TRA cohort, those with amenorrhea at 37 weeks were significantly older, had higher nodal status, and were more likely to be HR-positive. These investigators found no significant difference across the four treatment arms for rates of TRA in univariate analysis, and in multivariable analysis, the factors associated with higher risk of TRA were older age at diagnosis, the addition of taxanes to anthracycline-based chemotherapy, administration of docetaxel, carboplatin and trastuzumab treatment, and adjuvant ET use. With a median follow-up of 6.9 years, in the landmark analysis, a statistically significant interaction between TRA and HR status was found for DFS and OS.

Unlike the Lambertini study^4^, in B-47 we were able to examine the independent effects of chemotherapy on risk for amenorrhea, with and without the use of trastuzumab, and did not find that trastuzumab contributed to TRA. Rather, age, intended chemotherapy regimen, HR status, and ET use were associated with persistent amenorrhea at 36 months post randomization. As noted in the NSABP B-30 trial^2^, women receiving ET were more likely to remain amenorrheic than those without ET. Our landmark analysis of survival outcomes in the B-47 population was underpowered to examine the effects of TRA on these outcomes, and thus we cannot confirm the findings noted by Lambertini.

To the best of our knowledge, this is one of the largest prospective studies of menstrual function in premenopausal patients with breast cancer receiving contemporary adjuvant chemotherapy, with or without trastuzumab, including assessment of reproductive hormones and amenorrhea. This report provides much new information about the trajectory of TRA in younger patients with breast cancer and provides insight into the biology of TRA in this setting. Return of menses does not guarantee residual fertility, and more detailed information about the ovarian reserve is necessary to make clinical decisions about fertility in this patient population. Additional evaluation of some B-47 MH study participants that has been completed explores the added value of measuring anti-Mullerian hormone in this setting, to assess its role in understanding TRA^7^.

The findings from B-47 demonstrate the unreliability of amenorrhea in assessing menopausal status in younger breast cancer patients after adjuvant chemotherapy. This has implications for choice of ET, as well as considerations related to contraception. Laboratory assessment of estradiol and FSH should be performed to document menopausal status before considering initiation of aromatase inhibitors in this setting, because persistent amenorrhea to 36 months after treatment does not ensure postmenopausal status in premenopausal women.

## Methods

### MH study population

In addition to the main trial eligibility/exclusion criteria^1^, sub-study participant eligibility included: women with an intact uterus and at least one ovary were eligible if they had at least one menstrual period in the past 12 months; no use of current oral contraceptive hormones or other hormone replacement therapy. After written informed consent, eligible women were enrolled in the MH sub-study upon random assignment to the parent trial. Approximately 1500 eligible women were expected based on the planned total sample size.

### Treatments

Patients were randomly assigned to receive chemotherapy with or without trastuzumab, and were stratified by HER2 IHC score (1+ vs 2+), pathological nodal status (0–3, 4–9, ≥10), HR status (ER+ or PgR+ vs both negative), and intended chemotherapy regimen. Investigator choice between two chemotherapy regimens was declared upon study entry. An anthracycline (doxorubicin, cyclophosphamide followed by WP) or non-anthracycline regimen (docetaxel plus carboplatin) were the two options^1^. Patients received adjuvant radiotherapy and ET, as clinically indicated.

### Data collection

Study coordinators assessed MH status at baseline and at 3, 6, 12, 18, 24, 30, and 36 months of follow‐up using a standard questionnaire designed to assess menstrual bleeding history, any reported changes in menstrual cycle length, and history of hysterectomy and/or bilateral oophorectomy^2^. In the MH sub-study, consent was required for a blood specimen to measure reproductive hormones at baseline through 3, 6, 12, 18, and 24 months. To replicate assessments similar to routine clinical practice, blood samples were not timed to the menstrual cycle. Investigators were instructed to collect the sample within a one-week window surrounding the corresponding MH assessment with preference given to the same day. Baseline and follow‐up blood specimens were sent to a central clinical laboratory (Covance Laboratories, Indianapolis, IN) where standard laboratory assessment methodologies were used for estradiol and FSH. MH assessments and specimen collection were not continued for patients with an invasive breast cancer recurrence or second primary cancer.

ET use was assessed annually. If a patient reported any tamoxifen use during the time between randomization and the time point in question, then she was counted as a tamoxifen user. If any other ET medication was reported, she was included in an “other category.” All those with unknown ET status were excluded. For this analysis, a combination variable of HR status and ET use was constructed. The variable was defined with four categories: HR‐negative, HR‐positive with no ET use, HR‐positive with tamoxifen use, and HR‐positive with other use. Note that almost all the patients in the “other” category reported the use of aromatase inhibitors.

### Statistical considerations

Amenorrhea status was evaluated separately at the 12, 24, and 36‐month time points. Women whose last reported period was ≥6 months prior to the specific time point were categorized as amenorrheic. Patients who received a hysterectomy or bilateral oophorectomy prior to the specific time point were removed from the corresponding analyses. Amenorrhea status at each time point was compared between treatment groups using logistic regression. Logistic regression (univariable/multivariable) was used to examine other potential explanatory amenorrhea predictors including age, race, ethnicity, BMI, chemotherapy regimen, IHC score, number of positive nodes, HR status, histologic grade, and HR status/ET use. All comparisons were adjusted for age, as a known variable associated with amenorrhea. To investigate the prognostic effect of amenorrhea, we conducted a 12-month landmark Kaplan-Meier analysis for IDFS, stratified by treatment group, IHC score, number of positive nodes, HR status, and chemotherapy regimen.

Estradiol and FSH levels were dichotomized based on predetermined thresholds for identifying levels in postmenopausal ranges. An estradiol level of <20 pg/mL and an FSH level of >50 mIU/mL were used as cutoffs. The latter was chosen as a conservative measure of late menopausal transition as defined by the Stages of Reproductive Aging Workshop criteria^8^, for which amenorrhea >60 days and FSH > 25 define this stage. In addition, we examined the simultaneous occurrence of low estradiol and high FSH as a stronger indicator of loss of ovarian function. The dichotomized groups were then compared between treatment arms and other categories of explanatory variables as described above for amenorrhea. We also examined the relationships between hormone levels and TRA at both 12 and 24 months. A Cohen’s simple kappa statistic for the rate of agreement was calculated to determine whether amenorrheic patients were more likely to have hormone suppression, without regard to treatment assignment or other predictors.

The laboratory reported the lowest levels of estradiol as either <19 or <15 pg/mL, preventing exploration of mean values. However, FSH was reported as a continuous value. Thus, we explored mean FSH levels over time by treatment group, intended chemotherapy regimen, HR status/ET use, age, and BMI. Mixed model analyses with repeated measures were used to obtain least-square means which were plotted over time points.

Amenorrhea analyses included patients who completed the baseline MH questionnaire and at least one follow‐up questionnaire. Hormone analyses included patients with baseline samples and at least one follow‐up time point. All analyses followed an intention‐to‐treat principle for both trastuzumab and chemotherapy regimen. All assessments were based on a two-sided test with *α* of 0.05.

This multicenter trial was approved by the National Cancer Institute’s Central Institutional Review Board and/or local human investigations committees or institutional review boards at institutions participating in this multicenter trial in accordance with National Cancer Institute policies and procedures and assurances filed with and approved by the Department of Health and Human Services.

### Reporting summary

Further information on research design is available in the Nature Research Reporting Summary linked to this article.

## Supplementary information

GANZ: B-47 MH Suppl Materials

Reporting Summary

## Data Availability

The study protocol and informed consent form will be made available. Individual participant data that underlie the results reported in this article, after de-identification, will generally be available within one year after publication and will be accessible through the NCTN Data Archive. Data will be available to researchers who wish to analyze the data in secondary studies to enhance the public health benefit of the original work. Requirements may include (but not be limited to): a research plan, a Data Use Agreement (DUA), and legally binding signatures. https://nctn-data-archive.nci.nih.gov/. The data generated and analyzed during this study are described in the following data record: 10.6084/m9.figshare.14169878^9^.

## Code availability

Analyses were performed using SAS standard procedures (v9.4; SAS Institute, Cary, NC). Custom code or mathematical algorithms central to the conclusions were not used.
