# Supplementary material for: NRG Oncology/NSABP B-47 menstrual history study: impact of adjuvant chemotherapy with and without trastuzumab
Source: NPJ Breast Cancer. 2021 May 20;7:55. doi: 10.1038/s41523-021-00264-2 (PMC8137688; doi:10.1038/s41523-021-00264-2)
Supplement: Supplementary file 1 — GANZ: B-47 MH Suppl Materials [file 41523_2021_264_MOESM1_ESM.docx]

**Ganz, et al: NSABP B-47 Menstrual History**

**SUPPLEMETARY MATERIALS**

**TABLES**

1. **Supplementary Table 1.**

Association between Amenorrhea Status and Estradiol and FSH levels at 12 months and

24 months: NSABP B-47

**2. Supplementary Table 2.**

Multivariable Logistic Model Predicting Amenorrhea Status at 36 Months: NSABP B-47

1. **Supplementary Table 3.**

Results of Logistic Regression Predicting Estradiol Levels <20 pg/mL and FSH Levels ≥50 mIU/mL: NSABP B-47

**4. Supplementary Table 4.**

Persistent Amenorrhea at 24 and 36 Months: NSABP B-47

**FIGURES**

1. **Supplementary Figure 1.**

Rate of Amenorrhea at each Timepoint for Chemotherapy and Chemotherapy plus Trastuzumab Intention-to-treat Groups

Excludes those who had a hysterectomy/oophorectomy at each timepoint: NSABP B-47

1. **Supplementary Figure 2.**

Invasive Disease-free Survival by Amenorrhea Status. 12-month Landmark Analysis: NSABP B-47

**Supplementary Table 1.**

Association between amenorrhea status and estradiol and FSH levels at 12 months and 24 months: NSABP B-47

| Amenorrhea Status | Estradiol Level | | | | FSH Level | | | | Estradiol / FSH Level | | | | Total |
| --- | --- | --- | --- | --- | --- | --- | --- | --- | --- | --- | --- | --- | --- |
|  | ≥ 20 pg/mL | | <20 pg/mL | | <50 mIU/mL | | ≥ 50 mIU/mL | | Est ≥ 20 and/or FSH < 50 | | Est < 20 and FSH ≥ 50 | |  |
|  | No. | Row % | No. | Row % | No. | Row % | No. | Row % | No. | Row % | No. | Row % |  |
| At 12 Months |  |  |  |  |  |  |  |  |  |  |  |  |  |
|  |  |  |  |  |  |  |  |  |  |  |  |  |  |
| Not Amenorrheic | 101 | 71.6 | 40 | 28.4 | 127 | 90.1 | 14 | 9.9 | 133 | 94.3 | 8 | 5.7 | 141 |
|  |  |  |  |  |  |  |  |  |  |  |  |  |  |
| Amenorrheic | 166 | 22.5 | 573 | 77.5 | 476 | 64.4 | 263 | 35.6 | 525 | 71.0 | 214 | 29.0 | 739 |
|  |  |  |  |  |  |  |  |  |  |  |  |  |  |
| Total | 267 | 30.3 | 613 | 69.7 | 603 | 68.5 | 277 | 31.5 | 658 | 74.8 | 222 | 25.2 | 880 |
|  |  |  |  |  |  |  |  |  |  |  |  |  |  |
|  | Kappa: | | 0.36 | | Kappa: | | 0.11 | | Kappa: | | 0.09 | |  |
| At 24 Months |  |  |  |  |  |  |  |  |  |  |  |  |  |
|  |  |  |  |  |  |  |  |  |  |  |  |  |  |
| Not Amenorrheic | 98 | 78.4 | 27 | 21.6 | 116 | 92.8 | 9 | 7.2 | 119 | 95.2 | 6 | 4.8 | 125 |
|  |  |  |  |  |  |  |  |  |  |  |  |  |  |
| Amenorrheic | 118 | 22.6 | 405 | 77.4 | 391 | 74.8 | 132 | 25.2 | 410 | 78.4 | 132 | 25.2 | 523 |
|  |  |  |  |  |  |  |  |  |  |  |  |  |  |
| Total | 216 | 33.3 | 432 | 66.7 | 507 | 78.2 | 141 | 21.8 | 529 | 81.6 | 138 | 21.3 | 648 |
|  |  |  |  |  |  |  |  |  |  |  |  |  |  |
|  | Kappa: | | 0.44 | | Kappa: | | 0.08 | | Kappa: | | 0.08 | |  |

**Supplementary Table 2.**

Multivariable logistic model predicting amenorrhea status at 36 Months: NSABP B-47

| Characteristic | Multivariable Model at 36 Months | | |
| --- | --- | --- | --- |
|  | OR | 95% CI | *P* |
| Age at Entry | 1.31 | 1.26 - 1.36 | <.0001 |
| Chemotherapy Regimen |  |  |  |
| AC→WP | Ref |  | 0.02 |
| TC | 0.6 | 0.39 - 0.94 |  |
| HR Status/ET Use |  |  |  |
| HR - | Ref |  | 0.002 |
| HR +, none | 2.65 | 0.50 - 13.97 |  |
| HR +, tamoxifen | 2.23 | 1.33 - 3.72 |  |
| HR +, other | 10.47 | 2.44 - 44.95 |  |

AC🡪WP, doxorubicin and cyclophosphamide followed by weekly paclitaxel for 12 weeks; ET, endocrine therapy; HR, hormone receptor; TC, docetaxel plus cyclophosphamide.

**Supplementary Table 3**. Results of logistic regression^1^ predicting estradiol levels <20 pg/mL and FSH levels ≥50 mIU/mL: NSABP B-47

| Characteristic | 6 Months | | | | | 12 Months | | | | | 24 Months | | | | |
| --- | --- | --- | --- | --- | --- | --- | --- | --- | --- | --- | --- | --- | --- | --- | --- |
|  | No. of Pts | % Est<20 &FSH≥50 | Adj for Age & Baseline Est/FSH | | | No. of Pts | % Est<20 &FSH≥50 | Adj for Age & Baseline Est/FSH | | | No. of Pts | % Est<20 &FSH≥50 | Adj for Age & Baseline Est/FSH | | |
|  |  |  | OR | (95% CI) | P |  |  | OR | (95% CI) | P |  |  | OR | (95% CI) | P |
| Race |  |  |  |  |  |  |  |  |  |  |  |  |  |  |  |
| White | 821 | 54.0 | Ref |  | 0.47 | 820 | 29.1 | Ref |  | 0.23 | 649 | 23.6 | Ref |  | 0.16 |
| Black | 80 | 47.5 | 0.82 | 0.52-1.31 |  | 69 | 26.1 | 1.02 | 0.57-1.80 |  | 53 | 18.9 | 0.94 | 0.45-1.94 |  |
| Other | 50 | 46.0 | 0.75 | 0.42-1.34 |  | 54 | 16.7 | 0.52 | 0.25-1.10 |  | 43 | 9.3 | 0.36 | 0.13-1.03 |  |
| Ethnicity |  |  |  |  |  |  |  |  |  |  |  |  |  |  |  |
| Not Hispanic or Latino | 891 | 52.6 | Ref |  | 0.51 | 882 | 28.1 | Ref |  | 0.62 | 698 | 22.5 | Ref |  | 0.34 |
| Hispanic or Latino | 62 | 54.8 | 1.20 | 0.71-2.02 |  | 63 | 22.2 | 0.85 | 0.46-1.60 |  | 43 | 14.0 | 0.65 | 0.27-1.58 |  |
| Body Mass Index |  |  |  |  |  |  |  |  |  |  |  |  |  |  |  |
| <18.5 | 11 | 81.8 | 3.30 | 0.69-15.72 | <.0001 | 11 | 18.2 | 0.43 | 0.09-2.07 | <.0001 | 7 | 0.0 | *Not included* | | |
| 18.5-24.9 | 336 | 61.0 | Ref |  |  | 338 | 36.1 | Ref |  |  | 256 | 25.0 | Ref |  | 0.38 |
| 25.0-29.9 | 292 | 57.5 | 0.88 | 0.64-1.22 |  | 293 | 28.0 | 0.69 | 0.49-0.99 |  | 241 | 22.0 | 0.89 | 0.58-1.36 |  |
| ≥30.0 | 333 | 38.7 | 0.39 | 0.28-0.54 |  | 322 | 19.3 | 0.39 | 0.27-0.56 |  | 255 | 19.6 | 0.74 | 0.48-1.13 |  |
| Treatment |  |  |  |  |  |  |  |  |  |  |  |  |  |  |  |
| Chemo | 474 | 50.4 | Ref |  | 0.20 | 460 | 28.0 | Ref |  | 0.77 | 368 | 21.5 | Ref |  | 0.79 |
| Chemo + Trast | 498 | 54.6 | 1.18 | 0.92-1.53 |  | 504 | 27.6 | 0.96 | 0.72-1.28 |  | 391 | 22.5 | 1.05 | 0.74-1.49 |  |
| Intended Chemo Regimen |  |  |  |  |  |  |  |  |  |  |  |  |  |  |  |
| AC→WP | 681 | 54.6 | Ref |  | 0.008 | 668 | 27.5 | Ref |  | 0.46 | 531 | 22.8 | Ref |  | 0.12 |
| TC | 291 | 47.8 | 0.68 | 0.51-0.90 |  | 296 | 28.4 | 0.89 | 0.65-1.22 |  | 228 | 20.2 | 0.73 | 0.49-1.08 |  |
| IHC Score |  |  |  |  |  |  |  |  |  |  |  |  |  |  |  |
| 1+ | 552 | 56.5 | Ref |  | 0.004 | 550 | 27.5 | Ref |  | 0.71 | 436 | 22.5 | Ref |  | 0.73 |
| 2+ | 420 | 47.4 | 0.69 | 0.53-0.89 |  | 414 | 28.3 | 1.06 | 0.79-1.42 |  | 323 | 21.4 | 0.94 | 0.66-1.34 |  |
| Number of Positive Nodes |  |  |  |  |  |  |  |  |  |  |  |  |  |  |  |
| 0 | 183 | 52.5 | Ref |  | 0.31 | 171 | 33.9 | Ref |  | 0.14 | 139 | 25.9 | Ref |  | 0.31 |
| 1-3 | 520 | 52.9 | 0.98 | 0.70-1.38 |  | 538 | 26.4 | 0.65 | 0.44-0.96 |  | 418 | 20.1 | 0.71 | 0.45-1.13 |  |
| 4-9 | 198 | 55.6 | 1.09 | 0.72-1.64 |  | 188 | 27.7 | 0.70 | 0.44-1.12 |  | 150 | 21.3 | 0.78 | 0.44-1.36 |  |
| 10+ | 71 | 42.3 | 0.64 | 0.37-1.13 |  | 67 | 23.9 | 0.57 | 0.29-1.10 |  | 52 | 28.8 | 1.17 | 0.57-2.43 |  |
| Hormone Receptor Status |  |  |  |  |  |  |  |  |  |  |  |  |  |  |  |
| ER and PgR negative | 135 | 54.8 | Ref |  | 0.50 | 126 | 42.1 | Ref |  | <.0001 | 91 | 41.8 | Ref |  | <.0001 |
| ER and/or PgR positive | 837 | 52.2 | 0.88 | 0.61-1.28 |  | 838 | 25.7 | 0.43 | 0.29-0.64 |  | 668 | 19.3 | 0.32 | 0.20-0.51 |  |
| Histologic Grade |  |  |  |  |  |  |  |  |  |  |  |  |  |  |  |
| Low | 85 | 54.1 | Ref |  | 0.82 | 89 | 21.3 | Ref |  | 0.03 | 77 | 15.6 | Ref |  | 0.03 |
| Intermediate | 408 | 54.2 | 1.09 | 0.68-1.75 |  | 412 | 27.2 | 1.64 | 0.93-2.89 |  | 328 | 20.4 | 1.64 | 0.82-3.27 |  |
| High | 478 | 50.8 | 0.99 | 0.62-1.60 |  | 462 | 29.7 | 2.05 | 1.17-3.60 |  | 353 | 24.6 | 2.3 | 1.16-4.57 |  |
| HR Status/ET use^2^ | *Not Available* | | | | |  |  |  |  |  |  |  |  |  |  |
| HR- |  |  |  |  |  | 128 | 43.0 | Ref |  | <.0001 | 92 | 41.3 | Ref |  | <.0001 |
| HR+, none |  |  |  |  |  | 27 | 37.0 | 0.92 | 0.38-2.24 |  | 12 | 41.7 | 1.01 | 0.28-3.58 |  |
| HR+, tamoxifen |  |  |  |  |  | 702 | 20.7 | 0.32 | 0.21-0.49 |  | 580 | 14.3 | 0.24 | 0.15-0.39 |  |
| HR+, other |  |  |  |  |  | 107 | 54.2 | 1.27 | 0.74-2.17 |  | 75 | 54.7 | 1.49 | 0.79-2.83 |  |

^1^Each characteristic was assessed univariably in separate logistic regression models, however, all except age group were adjusted for continuous age upon random assignment.

^2^Endocrine therapy use in the first 6 months after random assignment was not available.

AC🡪WP, doxorubicin and cyclophosphamide followed by weekly paclitaxel for 12 weeks; ER, estrogen receptor; ET, endocrine therapy; HR, hormone receptor; IHC, immunohistochemistry; PgR, progesterone receptor; TC, docetaxel plus cyclophosphamide; Trast, trastuzumab.

**Supplementary Table 4.**

Persistent amenorrhea at 24 and 36 months: NSABP B-47

|  | Amenorrheic  at 12 Months | | Amenorrheic,  Estradiol<20, and FSH ≥50  at 12 Months | |
| --- | --- | --- | --- | --- |
|  | No. | % | No. | % |
| Amenorrhea Status at 24 Months |  |  |  |  |
| Amenorrheic | 847 | 79.0 | 180 | 84.1 |
| Not amenorrheic | 110 | 10.3 | 15 | 7.0 |
| Surgically induced | 65 | 6.1 | 8 | 3.7 |
| Unknown | 50 | 4.7 | 11 | 5.1 |
| Amenorrhea Status at 36 Months |  |  |  |  |
| Amenorrheic | 750 | 70.0 | 160 | 74.8 |
| Not amenorrheic | 103 | 9.6 | 15 | 7.0 |
| Surgically induced | 99 | 9.2 | 12 | 5.6 |
| Unknown | 120 | 11.2 | 27 | 12.6 |
| Total | 1,072 | 100.0 | 214 | 100.0 |

**Supplementary Figure 1.**

Rate of amenorrhea at each timepoint for chemotherapy and chemotherapy plus trastuzumab intention-to-treat groups: NSABP B-47

Excludes those who had a hysterectomy/oophorectomy at each timepoint

**Supplementary Figure 2.**

Invasive disease-free survival by amenorrhea status. 12-month landmark analysis: NSABP B-47

**
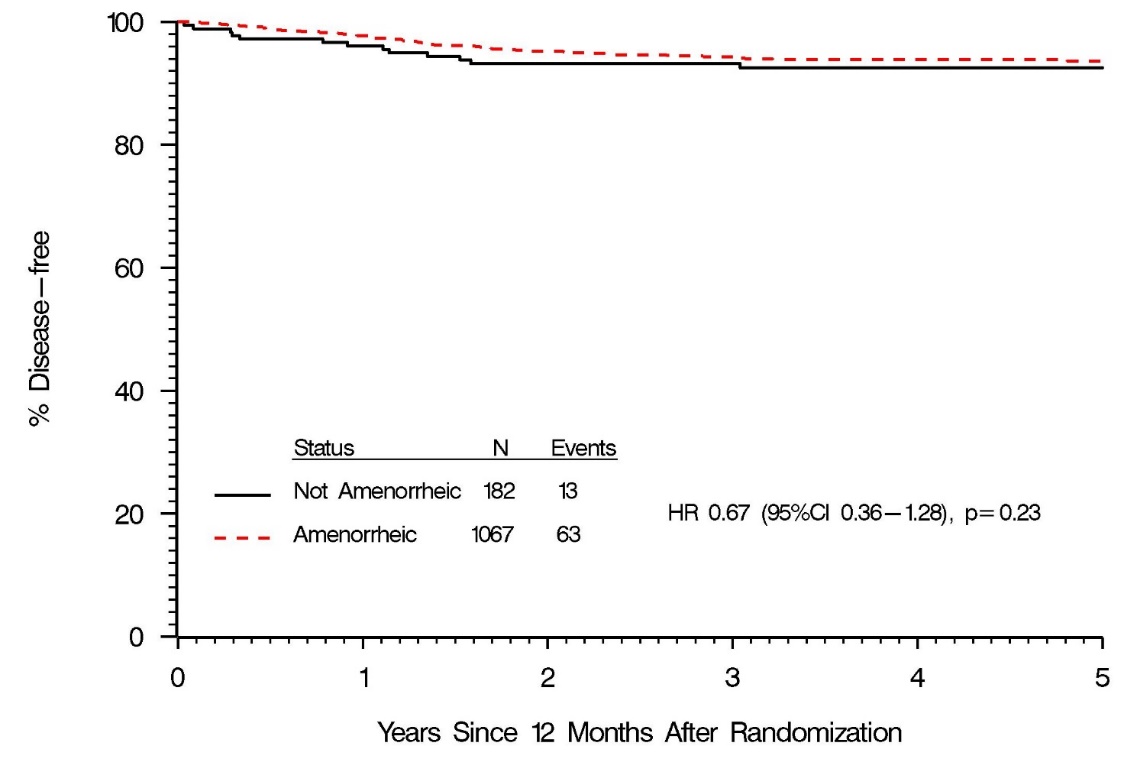
**
