# Supplementary material for: NRG Oncology/NSABP B-47 menstrual history study: impact of adjuvant chemotherapy with and without trastuzumab
Source: NPJ Breast Cancer. 2021 May 20;7:55. doi: 10.1038/s41523-021-00264-2 (PMC8137688; doi:10.1038/s41523-021-00264-2)
Supplement: Supplementary file 2 — Reporting Summary [file 41523_2021_264_MOESM2_ESM.pdf]

nature research

Corresponding author(s): Patricia A. Ganz, MD

Last updated by author(s): Feb 26, 2021

Reporting Summary

Nature Research wishes to improve the reproducibility of the work that we publish. This form provides structure for consistency and transparency in reporting. For further information on Nature Research policies, see our Editorial Policies and the Editorial Policy Checklist.

Statistics

For all statistical analyses, confirm that the following items are present in the figure legend, table legend, main text, or Methods section.

n/a	Confirmed	
☐	☒	The exact sample size (*n*) for each experimental group/condition, given as a discrete number and unit of measurement
☐	☒	A statement on whether measurements were taken from distinct samples or whether the same sample was measured repeatedly
☐	☒	The statistical test(s) used AND whether they are one- or two-sided<br>*Only common tests should be described solely by name; describe more complex techniques in the Methods section.*
☐	☒	A description of all covariates tested
☐	☒	A description of any assumptions or corrections, such as tests of normality and adjustment for multiple comparisons
☐	☒	A full description of the statistical parameters including central tendency (e.g. means) or other basic estimates (e.g. regression coefficient) AND variation (e.g. standard deviation) or associated estimates of uncertainty (e.g. confidence intervals)
☒	☐	For null hypothesis testing, the test statistic (e.g. *F*, *t*, *r*) with confidence intervals, effect sizes, degrees of freedom and *P* value noted<br>*Give P values as exact values whenever suitable.*
☒	☐	For Bayesian analysis, information on the choice of priors and Markov chain Monte Carlo settings
☒	☐	For hierarchical and complex designs, identification of the appropriate level for tests and full reporting of outcomes
☒	☐	Estimates of effect sizes (e.g. Cohen's *d*, Pearson's *r*), indicating how they were calculated

Our web collection on statistics for biologists contains articles on many of the points above.

Software and code

Policy information about availability of computer code

Data collection	No software was used for data collection
Data analysis	All analyses were performed using SAS (v9.4; SAS Institute, Cary, NC).

For manuscripts utilizing custom algorithms or software that are central to the research but not yet described in published literature, software must be made available to editors and reviewers. We strongly encourage code deposition in a community repository (e.g. GitHub). See the Nature Research guidelines for submitting code & software for further information.

Data

Policy information about availability of data

All manuscripts must include a data availability statement. This statement should provide the following information, where applicable:

- Accession codes, unique identifiers, or web links for publicly available datasets
- A list of figures that have associated raw data
- A description of any restrictions on data availability

Individual participant data that underlie the results reported in this article, after de-identification, will generally be available within one year after publication and will be accessible through the NCTN Data Archive. Data will be available to researchers who wish to analyze the data in secondary studies to enhance the public health benefit of the original work. Requirements may include (but not be limited to): a research plan, a Data Use Agreement (DUA), and legally binding signatures. https://nctn-data-archive.nci.nih.gov/

Field-specific reporting

Please select the one below that is the best fit for your research. If you are not sure, read the appropriate sections before making your selection.

☐ Life sciences ☒ Behavioural & social sciences ☐ Ecological, evolutionary & environmental sciences

For a reference copy of the document with all sections, see nature.com/documents/nr-reporting-summary-flat.pdf

Behavioural & social sciences study design

All studies must disclose on these points even when the disclosure is negative.

Study description	This report is of a menstrual history sub-study of a phase III, multicenter, randomized adjuvant therapy trial designed to evaluate the addition of trastuzumab to adjuvant chemotherapy in patients with HER2-low breast cancer. The primary aim of the substudy was to assemble an observational cohort of pre/perimenopausal women to evaluate the effect of trastuzumab on treatment-related amenorrhea (TRA)and the associations between TRA and circulating reproductive hormone levels.
Research sample	In addition to the main B-47 trial eligibility/exclusion criteria, sub-study participant eligibility included: women with an intact uterus and at least one ovary were eligible if they had at least one menstrual period in the past 12 months; no use of current oral contraceptive hormones or other hormone replacement therapy.
Sampling strategy	All B-47 trial participants were assessed for eligibility to the MH sub-study. Approximately 1500 eligible women were expected based on the planned total sample size of the parent trial.
Data collection	Study coordinators assessed MH status using a standard questionnaire designed to assess menstrual bleeding history, any reported changes in menstrual cycle length, and history of hysterectomy and/or bilateral oophorectomy. Blood samples were not timed to the menstrual cycle and were collected within a one-week window surrounding the corresponding MH assessment.
Timing	MH questionnaires were completed at baseline and at 3, 6, 12, 18, 24, 30, and 36 months of follow-up. Blood specimens were collected at baseline and at 3, 6, 12, 18, and 24 months.
Data exclusions	All valid data collected were used for the analyses.
Non-participation	The MH sub-study enrolled 1,458 eligible women, and 1,231 consented to blood sample collection. Both baseline and at least one follow-up MH form were available for 1,428 patients. Both baseline and at least one follow-up blood hormone measure were available for 1,123 patients.
Randomization	Patients were randomly assigned to receive chemotherapy with or without trastuzumab, and were stratified by HER2 IHC score (1+ vs 2+), pathological nodal status (0-3, 4-9, ≥10), hormone receptor status (ER+ or PgR+ vs both negative), and intended chemotherapy regimen.

Reporting for specific materials, systems and methods

We require information from authors about some types of materials, experimental systems and methods used in many studies. Here, indicate whether each material, system or method listed is relevant to your study. If you are not sure if a list item applies to your research, read the appropriate section before selecting a response.

Materials & experimental systems

n/a	Involved in the study	
☒	☐	Antibodies
☒	☐	Eukaryotic cell lines
☒	☐	Palaeontology and archaeology
☒	☐	Animals and other organisms
☐	☒	Human research participants
☐	☒	Clinical data
☒	☐	Dual use research of concern

Methods

n/a	Involved in the study	
☒	☐	ChIP-seq
☒	☐	Flow cytometry
☒	☐	MRI-based neuroimaging

Human research participants

Policy information about studies involving human research participants

Population characteristics	Mean age of participants was 44.1 years. 81.5% were White, 90.5% were Non-hispanic, and 86.1% were estrogen receptor and/or progesterone receptor positive.
Recruitment	Eligible women were enrolled in the B-47 MH substudy upon random assignment to the parent trial.

Ethics oversight	This multicenter trial was approved by the National Cancer Institute's Central Institutional Review Board (CIRB) and/or local human investigations committees or institutional review boards at institutions participating in this multicenter trial in accordance with National Cancer Institute policies and procedures and assurances filed with and approved by the Department of Health and Human Services. Written informed consent was required for participation.

Note that full information on the approval of the study protocol must also be provided in the manuscript.

Clinical data

Policy information about clinical studies

All manuscripts should comply with the ICMJE guidelines for publication of clinical research and a completed CONSORT checklist must be included with all submissions.

Clinical trial registration	Clinical Trials.gov: NCT01275677
Study protocol	The study protocol and informed consent form will be made available.
Data collection	The B-47 protocol entered 3,270 patients between February 8, 2011 and February 10, 2015. The MH sub-study enrolled 1,458 eligible women, and 1,231 consented to blood sample collection.
Outcomes	The primary aims of the menstrual history (MH) sub-study were to assemble an observational cohort of pre/perimenopausal women to evaluate the effect of trastuzumab on treatment related amenorrhea (TRA) and the associations between TRA and circulating reproductive hormone levels.
